# Synthesis of N-Doped Few-Layer Graphene through Shock-Induced Carbon Fixation from CO_2_

**DOI:** 10.3390/nano13010109

**Published:** 2022-12-26

**Authors:** Hao Yin, Xin Gao, Jianjun Liu, Pengwan Chen

**Affiliations:** 1 Institute of Systems Engineering, China Academy of Engineering Physics, Mianyang 612900, China; 2 State Key Laboratory of Explosion Science and Technology, Beijing Institute of Technology, Beijing 100081, China; 3 Advanced Technology Research Institute, Beijing Institute of Technology, Jinan 250307, China; 4 State Key Laboratory of Chemical Resource Engineering, Beijing University of Chemical Technology, Beijing 100029, China

**Keywords:** few-layer graphene, shock synthesis, nitrogen doping, CO_2_ conversion

## Abstract

In this study, graphene and N-doped graphene nanosheets were synthesized through the shock-induced reduction of CO_2_ using a cylindrical shock-loading apparatus. The mixture of solid CO_2_ and Mg powder was filled in the pre-cooled sample tube and then impacted by a shock-driven cylindrical flyer tube. The impact generated a shockwave that propagated into the mixed precursor, inducing a chemical reaction between CO_2_ and Mg at a high shock pressure and high shock temperature. The recovered black powders were characterized via various techniques, confirming the presences of few-layer graphene. The mechanism is carefully shown to be that CO_2_ was reduced by Mg to form few-layer graphene under shock-induced high pressure and high temperature. By adding carbamide as an N source, this synthetic route was also applied to synthesize N-doped graphene nanosheets. Moreover, the yield and mass of the graphene materials in this study are up to 40% and 0.5 g, respectively. This study showed an efficient and easy-to-scale-up route to prepare few-layer graphene and N-doped few-layer graphene through shock synthesis.

## 1. Introduction

Graphene, the first two-dimensional (2D) material synthesized by Noveselov et al. [1], has been proven to be an excellent future material [2,3,4,5,6,7,8]. Compared with other carbon nanomaterials, its atom-thick graphitic layer leads to its quantum confinement effect and contributes to its various unique properties [9], including outstanding mechanical properties [5,6,10], ultra-high thermal transport property [11,12], excellent optical properties adjusted by layers [13,14], superconductivity [15,16], electronic properties [1,4,17,18,19], etc. A vast number of research has revealed the abundant applications of graphene in multiple areas, such as field effect transistors [1,20], nano-sensors [21,22], metal-free electrodes [23,24], energy storage materials [25], biomedical carriers [26], etc. In addition, the properties of graphene materials can be adjusted and improved by their specific morphology [4,25], lateral dimension [27,28,29], elemental doping [30], and combination with other materials [31]. Among the large quantities of research on graphene, one central focus is to synthesize doped graphene materials with better electrochemical properties [30,31,32,33,34,35,36,37]. For example, the doped effect of phosphorus-doped rGO [38] and sulfur-doped rGO [39] allow graphene films to possess better capacities and higher rate performance because the doping increases the defect density of graphene and its carrier quantity.

Multiple approaches have been documented for producing graphene materials, including the mechanical exfoliation approach, the oxidation reduction method, epitaxial crystal growth, chemical vapor deposition (CVD), the liquid exfoliation method, the arc-discharge method, the electrochemical exfoliation method, pulsed wire discharge, etc. Through the mechanical exfoliation of highly oriented pyrolytic graphite, mono-layer graphene with a lateral size of 1 mm was obtained successfully [3,12]. In the oxidation reduction method, graphite flakes were partially oxidized to enlarge the graphitic layer distance and then exfoliated and reduced to form a reduced graphene oxide [8,40]. With respect to the epitaxial crystal growth method, the anneal treatment of SiC substrate in vacuum induces graphitization on its surface, which is identified as epitaxial graphene [41]. In the CVD method, the free carbon atoms from the hydrocarbon gases decomposition are transferred to deposit on certain metal surface to form graphene film [42]. Hernandez et al. [43] prepared a graphene suspension through the ultrasonic treatment on the graphite flakes dispersed in N-methylpyrrolidone by the ultrasonic cavitation phenomenon. Moreover, Geng et al. [44] mixed FeCl_3_ intercalated graphite powder with H_2_O_2_ to induce the decomposition of H_2_O_2_ to form O_2_ and the further expansion of graphitic layers to form graphene. In the arc-discharge method, Wu et al. [45] obtained high-quality graphene nanosheets via self-maintained direct current arc-discharge between two graphite electrodes in a mixture of inert gases. In these conditions, the graphite electrodes were ablated by the arc to generate free carbon atoms which were deposited on the chamber wall to form graphene. Rao et al. [46] applied an electrochemical reaction to prepare graphene materials through a primary battery consisting of two graphite rods and a salt solution, in which the electrochemical reaction induced the oxidation of graphitic layers and the intercalation of ions to exfoliate graphene. Through the pulsed wire discharge method, a graphite stick was exfoliated at a high inner pressure and high temperature induced by the rapid Joule heating of a pulsed discharge, leading to the formation of graphene [47,48].

In addition, the shock synthesis method can also be utilized to produce graphene [49]. In the shock synthesis method, under shock treatment, shock-induced transient high pressure and high temperature lead to intense chemical reaction resulting in the materials synthesis. Under these extreme conditions, a series of catastrophic changes occur in the chemical and physical properties of the materials. Various studies have been conducted using this process for material synthesis and modification. In particular, this unique process can form a supercritical reaction environment in microseconds, converting CO_2_ to useful carbon nanostructures. However, in a previous study, the yield of graphene recovered from shock-induced chemical reaction was 10%, approximately, due to the high sublimation rate of dry ice [50].

In this work, we optimized the shock synthesis method to prepare few-layer graphene (FLG) and N-doped FLG by using CO_2_, Mg fine powder, and carbamide (CH_4_N_2_O) with a high graphene yield. This work implies a potential large-scale production of graphene materials through carbon fixation using the shock synthesis method.

## 2. Materials and Methods

**Figure 1:**
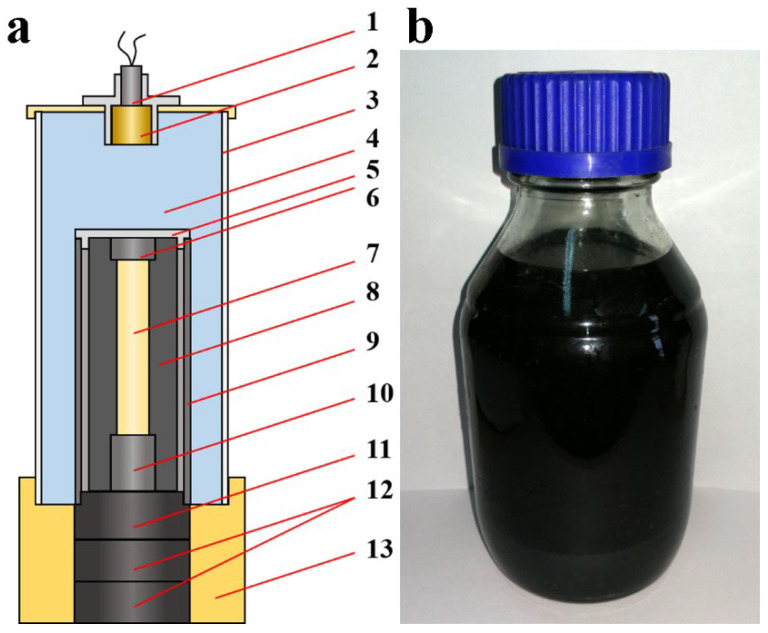
(**a**) Illustration of cylindrical shock recovery apparatus with flyer tube and (**b**) photograph of recovered graphene suspension after purification. 1—detonator; 2—RDX (cyclotrimethylenetrinitramine with detonation velocity of 8750 m/s); 3—PVC tube; 4—main charge; 5—support ring lid; 6—steel block; 7—sample; 8—sample tube; 9—cylindrical flyer tube; 10—steel block; 11—steel base; 12—momentum block; 13—plastic support.

To perform the shock treatment on precursors and for the recovery of the graphite samples, a cylindrical shock recovery apparatus was applied, as shown in Figure 1. The copper sample tube (10 mm in inner diameter and 100 mm length) was filled with mixed precursor powder and blocked using a copper plug. Then, the sample tube was immersed into liquid nitrogen for 30 min to inhibit the sublimation of solid CO_2_. Subsequently, the sample tube and cylindrical flyer tube were fixed on the cylindrical steel base with accessorial fixation of ring-like support to keep a gap of 2 mm between the sample tube and flyer tube. Then, the apparatus was assembled according to Figure 1a with the main charge (ammonium nitrate and fuel oil explosive, ANFO) for the shock treatment. In the apparatus, the cylindrical flyer tube can protect the main charge from the low temperature of sample tube to ensure stable detonation. During the detonation process, the flyer tube was accelerated by detonation to impact on the sample tube. The generated converging shockwave was transmitted into the cylindrical sample tube and acted on the precursor powder. Consequently, the shockwave front propagated in the mixed precursor powder with a conical wave front. This further enhanced the shock pressure through oblique impact [51], leading to multiple extreme effects on the precursor powder, such as transient high pressure and temperature, strong shear effect, etc. The incident shock pressure (P) and shock temperature (T) on the sample were calculated based on the Hugoniot curve of powder materials and the Mie–Grüneisen equation (as illustrated in literature [48,49]). After shock treatment, the sample was recovered and purified using 15% HCl (4.4 mol/L) to remove MgO powder and a small amount of Cu fragments from the copper sample tube. Finally, the sample suspension was washed to remove MgCl_2_ and HCl, and dried using a vacuum-frozen drier for further characterization.

In these shock synthesis experiments, the mixed precursor consists of solid CO_2_ as an oxidant and carbon source, Mg as a reductant, and carbamide as a nitrogen source. The solid CO_2_ and Mg powder were mixed at a molar ratio of 1.1:2 in a mixing chamber cooled by liquid nitrogen. For the experiment to synthesize N-doped graphene, the carbamide is added in the precursor as a nitrogen source with the molar ratio 1:0.1 (solid CO_2_:carbamide). The carbamide powder was firstly mixed with Mg powder and then subsequently mixed with solid CO_2_ in the mixing chamber cooled by liquid nitrogen. The mass of the sample tube before and after filling it with the precursor were measured to obtain the mass of precursor powder (m_0_). The detailed experimental conditions and calculated shock pressure were listed in Table 1.

An X-ray Diffractometer (XRD) (D/MAX-2500, Rikagu, Tokyo, Japan) was applied to record the XRD patterns of recovered samples with Cu Kα radiation (k = 0.15406 nm) and a step size of 0.0330° (2θ) from 10–90°. Transmission electron microscopy (TEM) and high-resolution TEM (HRTEM) observations of recovered samples were performed with a FEI Tecnai G^2^ F20 S-Twin transmission electron microscope at an accelerating voltage of 200 kV to analyze the micromorphology of the recovered samples. Field emission scanning electron microscopy (SEM) observations were performed using a Hitachi S-4800 at an accelerating voltage of 5–15 kV. A LabRAM Aramis Raman spectrometer equipped with a He–Ne laser (excitation wavelength of 633 nm) was carried out to record the Raman spectra of recovered samples. The chemical composition and bonding states of recovered samples were probed by X-ray photoelectron spectroscopy (XPS) using a Thermo ESCALAB 250 Xi spectrometer with monochromatic AlK (1486.6 eV) X-ray sources. The recorded XPS spectra of all recovered samples were fit using a Gaussian–Lorentzian peak with non-linear Shirley background correlation. In addition, the samples for the characterization of SEM and Raman were separated in distilled water via ultrasonic dispersion and then dried on a Si plate.

## 3. Results

**Figure 2:**
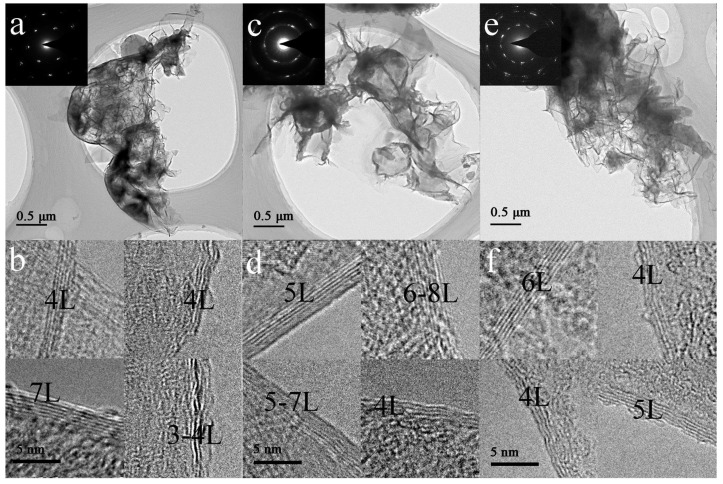
TEM and HRTEM images of recovered samples: (**a**) TEM and (**b**) HRTEM images of No. 1 sample, (**c**) TEM and (**d**) HRTEM images of No. 2 sample, (**e**) TEM and (**f**) HRTEM images of No. 3 sample.

Figure 2 presents the representative TEM and HRTEM images of recovered samples Nos. 1–3, indicating the presence of wrinkled and extended ultra-thin films with lateral sizes of 1~5 μm, which are in accordance with the typical micromorphology of graphene materials [40,41,42,43,44]. In addition, the edge observation of these ultra-thin films from HRTEM images (Figure 2b,d,f) further reveal that the layers of shock-synthesized ultra-thin films are in the range of 3–7, 4–8, and 4–6, respectively, with corresponding interlayer distances of 0.3–0.4 nm. Thus, the recovered samples are identified as FLG. Furthermore, the thicknesses of all three recovered samples are of the same order, implying that the experimental conditions induce similar formation conditions of FLG. Furthermore, the selected area electron diffraction (SAED) patterns (the insets of Figure 2a,c) of the corresponding samples display ring-like diffraction patterns with dispersed bright spots, suggesting the presence of representative rotational staking faults in the matrix of the graphene sheets [47,52]. This finding suggests that the high crystallinity of recovered FLG samples are in accordance with the results observed in highly crystalline FLG [48,50]. Moreover, the inset of Figure 2e shows a ring-like diffraction pattern with multiple dispersed spots, indicating that the crystallinity of No. 3 sample is partially disorientated due to the structural distortion caused by the interposition of N atoms [53]. Thus, the TEM examination shows that the FLG synthesized through shock loading process is highly crystalline.

**Figure 3:**
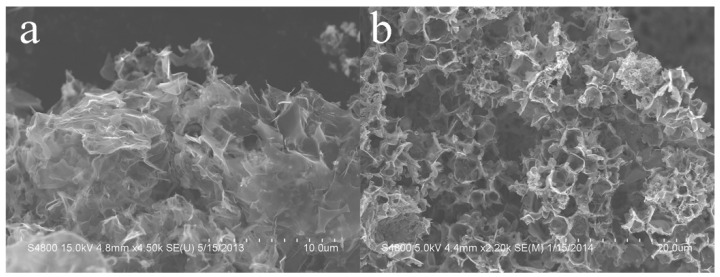
SEM images of (**a**) sample No. 1 and (**b**) sample No. 3.

The representative SEM images (Figure 3) of the FLG synthesized through the shock loading process indicate that the samples possess a typical micromorphology of 2D materials and curved and extended ultra-thin nanosheets [41,44,54] due to their thermodynamic instability [55]. Moreover, the formed graphene nanosheets agglomerate to form a 3D-porous-like structure, implying the thermodynamic instability of 2D materials. Furthermore, Figure 3 also reveals the presence of bowl-like FLG, which may imply that the MgO particles formed by shock-induced chemical reactions become the deposition substrate of reduced carbon atoms. Similar formation processes have been reported by Xu et al. [56] in the study on the formation of porous carbon nanomeshes using a self-sustained sol-pyrolysis approach. After the purification process, the MgO substrates are removed from the products. In addition, the lateral size distributions of recovered FLG are in the range of 1~5 μm, approximately, based on the statistics of SEM examination (30 SEM images for each sample).

**Figure 4:**
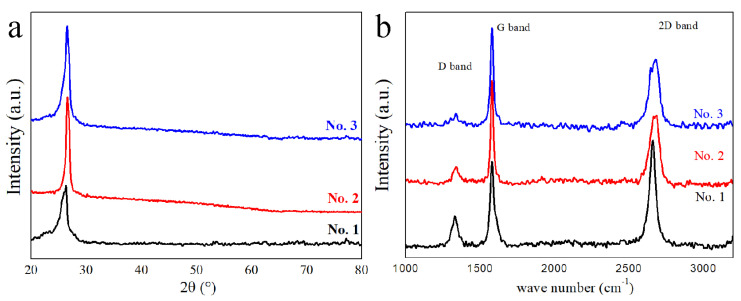
(**a**) XRD patterns and (**b**) Raman spectra of recovered samples.

Figure 4a shows the XRD patterns of the recovered samples, suggesting only one peak appearing at approximately 26.5° assigned to graphite (002) diffraction. It also demonstrates the existence of pure FLG in all recovered samples. The calculated lattice distance values corresponding to the (002) peaks of recovered samples are in range of 0.34–0.345 nm based on Bragg’s law. The calculated results are slightly larger than those of bulk graphite (0.335 nm), which is also in a good agreement with the TEM results and is in accordance with previous reports [23,48], thus showing the presence of FLG. Furthermore, the absence of XRD peaks corresponding to copper, MgO, MgCl_2_, etc., also indicates the high purity of the recovered FLG samples after the purification process.

The analysis based on Raman spectra is widely used as a facile and credible characterization of the phase and structure of carbon materials. Figure 4b presents the Raman spectra of recovered samples, in which three characteristic Raman bands of SP^2^ carbon are observed, including the D band (1332 cm^−1^), G band (1581 cm^−1^), and 2D band (in a range of 2663~2687 cm^−1^) in each spectrum. Through the spectra, the intensity ratios of the 2D band to the G band (I_2D_/I_G_) and the D band to the G band (I_D_/I_G_) are calculated to estimate the graphitic layers of the recovered samples [55,57] and the degree of disorder in the formed graphene nanosheets [58,59], respectively. The I_2D_/I_G_ values of the recovered samples are 0.71–1.13 (see Table 2) smaller than 2, indicating the presence of FLG [55,57] in the recovered samples. Note that the I_D_/I_G_ value of sample No. 1 is approximately 0.35, suggesting a high degree of disorder in the recovered graphene nanosheets. However, the I_D_/I_G_ values of samples No. 2 and 3 are in a range of 0.13–0.15 (see Table 2), suggesting the low degree of disorder in the recovered graphene nanosheets. These results may imply that the higher temperature and pressure condition contributes to the crystallinity of formed FLG nanosheets, which is in accordance with the results reported by Cheng et al. [60] on the Joule heating treatment on graphene films.

**Figure 5:**
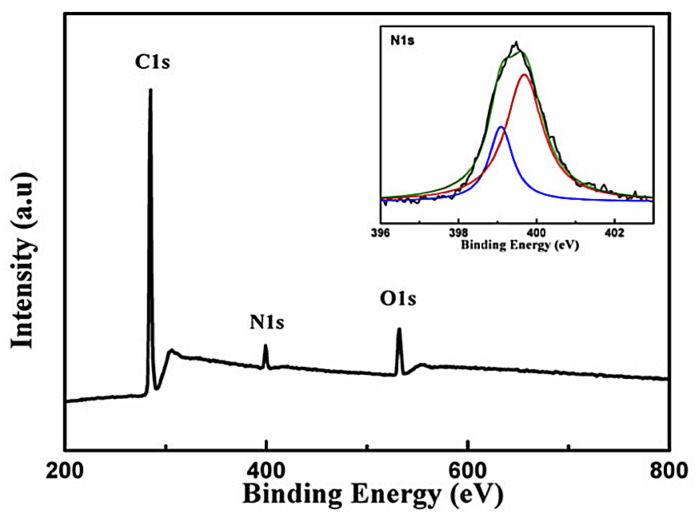
XPS spectrum of sample No. 3, the shock-synthesized N-doped graphene. Inset of Figure 5 presents the high-resolution N1s spectrum of sample No. 3.

XPS measurements were carried out to probe nitrogen atoms in sample No. 3 obtained from the precursor mixed with a nitrogen source, carbamide. As shown in Figure 5, the XPS spectrum of sample No. 3 contains an intense C1s band at ca. 284 eV, an N1s band at ca. 400 eV, and an O1s band at ca. 532 eV. Based on the band areas and atomic sensitivity factor of C1s and N1s, the corresponding N/C atomic ratio of sample No. 3 is calculated to be 6.71%. Note that the physical and chemical performance of doped graphene is positively correlated to the doping content [49,50]. Thus, this study further indicates that the transient extreme conditions generated by the shock loading process could benefit the atomic ratio of N/C, which provides an important basis for a wider application range of N-doped graphene. Furthermore, the N content is higher compared to previous studies, implying the higher chemical activity of synthesized graphene [49,50]. The high-resolution XPS N1s spectrum (inset of Figure 5) reveals two types of N-doping in the shock-synthesized graphene’s molecular structure, including pyridinic-like (399.1 eV) and pyrrolic-like (399.7 eV) N atoms [61]. Consequently, N atoms have been incorporated into the graphene hexagon rings of the N-doped graphene in sample No. 3. However, the O1s band appearing at 532 nm in the XPS spectrum is possibly due to the incorporation of physically adsorbed oxygen [50]. XPS analysis reveals that the atomic percentage of oxygen in the shock-synthesized N-doped graphene is 6.74 at%. Moreover, XPS results suggest that no extra bands of impure elements appeared other than those of C, N, and O, indicating the high purity of the N-doped graphene after the HCl purification process.

## 4. Discussion

The above characterizations confirm that shockwave action is an effective method to produce graphene materials. The formation mechanism of FLG from shock-treated precursor powder consisting of a mixture of solid CO_2_ and Mg is difficult to investigated via the in situ observation route due to the transient non-equilibrium process. We speculate that the formation of graphene materials in these experiments to be a shock-induced, dense fluid–solid reaction process. Considering that solid CO_2_ can react with Mg powder under high temperatures at atmospheric pressure, a similar chemical reaction can be easily ignited under the conditions of shock-induced high temperature (3000~4000 K) and high pressure (14~18 GPa). These extreme conditions sublimate solid CO_2_ rapidly to form a high-density CO_2_ fluid in an enclosed sample tube with a small amount of CO and O_2_ formed by the partial decomposition of CO_2_. The high density of CO_2_ fluid also contributes to the reaction rate of the mixed precursor. Furthermore, shockwave propagation through the precursor powder is much higher than the combustion rate of Mg and CO_2_, leading to the ultra-high reaction rate of the mixed precursor in microseconds. Under this condition, the reduced free C atoms and oxidized MgO separate uniformly in the sample tube. Consequently, the free C atoms deposit on the surface of the adjacent MgO powder to form few-layer graphitic layers during the subsequent ultra-fast cooling process. After the purification process, the few-layer graphitic layers remain as FLG.

Moreover, the ultra-fast reaction rate, led by the shock loading and the uniform separation of free C atoms and MgO powder, also contributes to the uniform deposition to form FLG with the same orders of thickness and lateral size. Note that the higher pressure and higher temperature also contribute to the crystallization process of graphene to form graphene with a higher crystallinity considering the transient duration of this crystallization process. With respect to sample No. 3, the carbamide molecules decompose to produce free C atoms and N atoms under extreme high pressure and temperature during shock loading. These C atoms and N atoms also deposit on MgO surface, forming graphene and inducing the nitrogen doping during the deposition process to form N-doped graphene.

In addition, the pre-cooling treatment of the sample tube using liquid nitrogen efficiently inhibits the CO_2_’s sublimation rate during experimental operation before detonation, leading to a high-efficiency preparation of graphene materials with a higher yield compared with previous studies. The graphene yield of samples Nos. 1–3 are in the range of approximately 70~80%. The formed N-doped graphene powder may have potential applications in the preparation of outstanding metal-free electrodes, which has been investigated using the specimen synthesized by the detonation method in our previous research.

## 5. Conclusions

In this study, graphene and N-doped graphene materials are obtained through the shock-induced chemical reaction of CO_2_ and Mg powder (with carbamide as N source). The shockwave technique provides an efficient carbon fixation route to transform CO_2_ into useful graphene materials. Experimental results confirm that shockwave action can induce the sublimation of solid CO_2_ to form dense CO_2_ fluid and further trigger the redox reaction between CO_2_ and Mg to produce FLG nanosheets within microseconds during the shock loading process. By adding carbamide as a N-doping agent, N-doped graphene can be obtained with a similar approach in one-step reaction route. The experimental results also imply that the increase in shock pressure and temperature is conducive to the crystallinity of the formed FLG and N-doped FLG though this shock-induced carbon fixation method.

## Figures and Tables

**Table 1:** Experimental conditions and the yield of shock-synthesized graphene *.

No.	Precursor Molar Ratio	*ρ*_00_ (g/cm^3^)	m_0_ (g)	v (km/s)	p (GPa)	T (K)	m_1_ (g)	Graphene Yield
1	CO_2_ + Mg 1.1:2	1.274	6.00	2.63	14.3	3170	4.73	78.8%
2		1.304	6.14	3.07	18.8	4010	4.36	70.1%
3	CO_2_ + Mg + carbamide 1.1:2:0.1	1.282	6.04	3.12	18.4	4470	4.80	79.5%

* *ρ*_00_ is packing density of mixed precursor powder, v is impact velocity, p is shock pressure, T is shock temperature, m_1_ is the mass of FLG sample after purification. Graphene yield is calculated according to the ratio of m_1_ to m_0_.

**Table 2:** Raman characterization results of recovered samples.

No.	I_2D_/I_G_	I_D_/I_G_	Main Phase
1	1.13	0.35	FLG
2	0.71	0.15	FLG
3	0.77	0.13	FLG

## Data Availability

Not applicable.
